# Aspirin vs Clopidogrel: Antiplatelet Agent of Choice for Those With Recent Bleeding or at Risk for Gastrointestinal Bleed

**DOI:** 10.7759/cureus.37890

**Published:** 2023-04-20

**Authors:** Siddharth Gosavi, Gokul Krishnan, Raviraja V Acharya

**Affiliations:** 1 Department of Internal Medicine, Kasturba Medical College Manipal, Manipal, IND

**Keywords:** proton pump inhibitor, endoscopy, gastrointestinal bleed, clopidogrel, aspirin

## Abstract

Antiplatelet agents are used worldwide mainly for primary and secondary prevention of cardiovascular events on a long-term basis for mortality benefit. Gastrointestinal bleeding is a well-known adverse effect. Various factors are to be considered while choosing antiplatelet agents to prevent the risk of bleed and rebleed incidents. These range from deciding on the agent, timing of therapy, underlying indications, coadministration of proton pump inhibitor, etc. At the same time, one must weigh the risks of cardiovascular events secondary to the stoppage of antiplatelet therapy. With this review, we have tried to guide the clinician on decision-making regarding the care of patients on management of acute upper and lower gastrointestinal bleeding, stoppage, restarting of agents, and measures to prevent a recurrence. We have focused on aspirin and clopidogrel as they are among the most widely used antiplatelet agents.

## Introduction and background

Antiplatelet agents are used worldwide for various indications. However, this is associated with the adverse effect of gastrointestinal bleeding. The pros and cons of agents are to be considered while initiating them. Existing literature shows the incidence of gastrointestinal bleed rates to be 0.7-1.3% for aspirin, 1.3% for clopidogrel, and 1.2-2% among those on dual antiplatelet therapy with aspirin and clopidogrel [1-3].

Han et al. performed a study on antiplatelet-induced gastrointestinal injury, where patients were randomised to receive aspirin plus placebo, clopidogrel plus placebo or dual antiplatelet therapy (DAPT) after six months of dual antiplatelet therapy. The incidence of bleeding at 12 months post-DAPT was 0% for aspirin, 1.2% for clopidogrel and 5.4% in the DAPT group [4].

The mortality rate in the bleeding group was 24.6% compared to 4.7% in the non-bleeding group [5]. Different agents have varying mechanisms of action. Aspirin acts by irreversible acetylation and inhibition of cyclooxygenase (COX) enzyme while clopidogrel causes competitive irreversible blockade of P2Y12. Other newer antiplatelet agents are reversible P2Y12 blockers (ticagrelor), GPIIb/IIIa inhibitors (abciximab) etc [6]. Regarding the indications of antiplatelet agents, they are prescribed for primary prevention and secondary prevention of cardiovascular diseases. They may be given as monotherapy or dual therapy. Various indications of platelet are mentioned in the table [7-9] (Table 1). 

**Table 1:** Indications of antiplatelet agents

Monotherapy	Dual antiplatelet therapy (DAPT)	Antiplatelet + anticoagulant
Primary prevention of cardiovascular diseases	Post coronary stenting for 12 months	Cardioembolic stroke
Secondary prevention of cardiovascular diseases	Post coronary artery bypass grafting(CABG)	Acute myocardial infarction
	NSTEMI	Atrial fibrillation with myocardial infarction
	Short-term following high-risk transient ischemic attack and post-ischemic cerebrovascular accident	
	Secondary prevention of cardiovascular disease involving polyvascular bed (≥ 2 vascular beds)	

There are a few scenarios where antiplatelet agents are combined with anticoagulants. In cases with high-risk atherosclerosis, combining low-dose rivaroxaban with aspirin can help reduce major cardiovascular events like myocardial infarction and cerebrovascular accidents [9]. Among those with indications for long-term anticoagulation like atrial fibrillation and venous thromboembolism, single or dual antiplatelet therapy may be added to ongoing anticoagulants temporarily. Considering the high risk of bleed associated with the combination of antiplatelets and anticoagulants, it is advised to give triple therapy for one week following coronary stenting for acute coronary syndrome and to decide on further management after assessing the risk of bleed and ischemia. If a patient is at high risk of ischemia, then triple therapy can be continued for one month followed by switching to dual therapy with a single antiplatelet and single anticoagulant. If the patient is at high risk of bleeding, then they should be switched to dual therapy within one week instead of one month [10-11].

With this review, we have tried to guide the clinician on minimizing the risk of antiplatelet-induced gastrointestinal bleeding, management of bleeding scenarios and how to go about restarting the agents. We have also focused on the factors to be considered for the prevention of antiplatelet-induced gastrointestinal bleeding in the future. The main focus of this review is on aspirin and clopidogrel which are the most widely used agents at present.

## Review

Mechanism and risk factors of antiplatelet-induced gastrointestinal bleed

­There are various mechanisms behind antiplatelet-induced gastrointestinal bleeding. Aspirin causes inhibition of protective COX enzymes while clopidogrel results in suppression of platelet-derived growth factor, reduced angiogenesis and delayed ulcer healing [12]. Apart from gastrointestinal bleeding, antiplatelet use is associated with bleed in other sites like intracerebral, surgical sites etc [13]. Common risk factors for antiplatelet-induced bleeding are mentioned in Table 2. The risk is 1.3% within the first 30 days of dual antiplatelet therapy [14]. Vascular ectasia is causative for about 4% of non-variceal upper GI haemorrhages [15].

**Table 2:** Risk factors for antiplatelet-induced gastrointestinal bleed

Risk factors
Dual antiplatelet therapy
Prolonged usage
Co-administration of anticoagulants
Other conditions predisposing the individual to bleed
Underlying peptic ulcer disease
Esophageal varices
Vascular ectasia

Drug interactions are to be considered if they could potentially increase the potency of antiplatelet agents and related bleeding. Potential interactions of some of the commonly used agents are mentioned for reference [16-18] (Table 3).

**Table 3:** Drug interactions with antiplatelet agents

Drug interactions with antiplatelet agents	
Antibiotic	
Amoxicillin	Increases aspirin levels by reducing renal clearance
Cefepime	Increases the effect of aspirin by competing for renal tubular clearance
Ceftazidime	Increases the effect of aspirin by competing for renal clearance
Piperacillin	Salicylic acid can be displaced from protein-binding sites
Other agents	
Hydrochlorothiazide	Increases the effect of aspirin by competing for renal clearance
Calcium carbonate	Calcium carbonate at moderate doses may cause aspirin toxicity while at higher doses it results in increased aspirin excretion

Aspirin vs clopidogrel

There are several studies comparing the gastrointestinal effects of aspirin and clopidogrel with conflicting reports [3,4,19-22] (Table 4). However, considering the possibility of interactions of clopidogrel with proton pump inhibitors (PPI), it may be better to consider aspirin over clopidogrel.

**Table 4:** Studies comparing the incidence of antiplatelet-induced gastrointestinal bleeding RCT: Randomised control trial; PPI: proton pump inhibitor; SAPT: Single antiplatelet therapy; DAPT: dual antiplatelet therapy

Reference	Type of study	Description of sample	Result
Aspirin-associated gastrointestinal bleed			
Chan et al., 2005 [19]	RCT	320 patients were enrolled among whom 161 received clopidogrel and 159 received aspirin plus esomeprazole	Aspirin plus esomeprazole was found to be superior to clopidogrel in the prevention of recurrent gastrointestinal ulcer bleeding. The incidence of ulcer bleeding was 8.6% in patients who received clopidogrel and 0.7% in patients who received aspirin and esomeprazole.
Hsiao et al., 2002 [20]	Retrospective cohort study	Cases over a period of 6 years who were hospitalised for gastrointestinal complications prior to initiation of antiplatelet agents were considered. It was done to assess the risk of recurrent gastrointestinal complications among those at high risk.	Aspirin plus PPI was associated with a reduced risk of major gastrointestinal complications compared to clopidogrel combined with PPI. The Incidence of hospitalisation for major gastrointestinal complications was 0.103 per person-year in the aspirin plus PPI group compared to 0.152 per person-year in the clopidogrel plus PPI group.
Clopidogrel associated with lesser gastrointestinal bleed			
CAPRIE trial, 1996 [21]	RCT	Those with evidence of atherosclerosis were randomised to receive aspirin or clopidogrel	Aspirin was associated with a higher risk of gastrointestinal bleeding compared to clopidogrel(0.72 % vs 0.52%) (p=0.05)
Aspirin and clopidogrel having equal effects with respect to gastrointestinal bleed			
Han et al., 2021 [4]	RCT	Those with no ulcerations or bleeding on capsule endoscopy done after 6 months of DAPT post-percutaneous intervention were randomised to receive aspirin plus placebo, clopidogrel plus placebo or aspirin plus clopidogrel. For an additional 6 months following which a repeat capsule endoscopy was done to look for gastrointestinal mucosal injury.	Aspirin and clopidogrel monotherapy had similar effects on the gastrointestinal mucosa
Ng et al. 2008 [22]	RCT	Those with aspirin-induced peptic ulcer disease treated with omeprazole were randomised to receive clopidogrel or low-dose aspirin	A prospective study among those who had developed peptic ulcers while on aspirin was randomised to receive aspirin plus PPI or clopidogrel PPI. Minor gastrointestinal bleeding occurred in 45% of the clopidogrel group compared to 42% in the aspirin group, however, it was not statistically significant (p=0.709).
SAPT vs DAPT			
Han et al., 2021 [4]	RCT	Those with no ulcerations or bleeding on capsule endoscopy done after 6 months of DAPT post-percutaneous intervention were randomised to receive aspirin plus placebo, clopidogrel plus placebo or aspirin plus clopidogrel. For an additional 6 months following which a repeat capsule endoscopy was done to look for gastrointestinal mucosal injury	SAPT causes lesser gastrointestinal injury compared to DAPT(68.1% vs 95.2%,p =0.006) Newer ulcers were higher in DAPT group (38.1%) compared to SAPT group (8.5%).
Diener et al., 2004 [3]	RCT	Those with recent ischemic stroke or transient ischemic attack and at least one additional vascular risk factor and already receiving clopidogrel 75 mg/day.were randomised to receive aspirin or a placebo in addition to clopidogrel.	Life-threatening bleed was higher in the DAPT group compared to clopidogrel monotherapy (2.6% vs 1.3%) (p<0.0001)

Management of upper gastrointestinal bleed

Cases of upper gastrointestinal bleed may not present with frank hematemesis and melena in all cases. In such scenarios, the following are to be looked for to suspect gastrointestinal bleeding drop in haemoglobin, reactive thrombocytosis and elevated urea. Patients already on antiplatelet agents who have an episode of upper gastrointestinal bleeding should be treated for their bleeding after stopping the agent and then when the time comes to resume the antiplatelet drugs the indications should be clearly looked into. If the patient is on antiplatelet agents for primary prevention, consider discontinuation of the antiplatelet agent.

If an antiplatelet agent was being taken for secondary prevention, then the practitioner should decide on further management on the basis of the risk of rebleed and underlying indication for antiplatelet therapy. If the patient is at low risk of rebleed, then antiplatelet agents can be resumed immediately after endoscopy. On the other hand, if the patient is at high risk of gastrointestinal bleed then the practitioner should look at the status of percutaneous transluminal coronary angioplasty (PTCA). If the bleed was within four weeks of bare metal stent or six months of a drug-eluting stent, then it is recommended to restart antiplatelet agents within three days in view of the high risk of stent thrombosis. If the bleed occurred beyond this period, then we can wait for up to five days after the last dosing. The risk of rebleeding can be assessed from endoscopy findings. Visible vessels and adherent clots are two findings suggestive of a high risk of rebleeding [23].

Supportive management is in the form of blood products, and parenteral fluids to be provided on basis of clinical assessment. Definitive management including proton pump infusion and endoscopy-guided interventions. Gastrointestinal endoscopy has the following roles in case of suspected antiplatelet-induced upper gastrointestinal bleed [24] (Table 5).

**Table 5:** Role of endoscopy in antiplatelet-induced gastrointestinal bleed

Role of endoscopy in antiplatelet-induced gastrointestinal bleed	
Diagnostic	Assess the risk of rebleed
	Confirm the bleed is due to an antiplatelet agent by ruling out other causes like variceal bleed
	The severity of ulcer (Forest grading etc)
	H. pylori testing
Therapeutic	Sclerotherapy, clipping

At present, there are no antidotes for rapid reversal like that for anticoagulants. Neurocritical care guidelines advise a single dose of desmopressin for antiplatelet-induced intracranial haemorrhages but not for gastrointestinal bleeding. Desmopressin acetate (DDAVP) improves platelet function by increasing the release of von Willebrand factor (vWF) and factor VIII from the endothelium [25].

Management of lower gastrointestinal bleed

**Figure 1:**
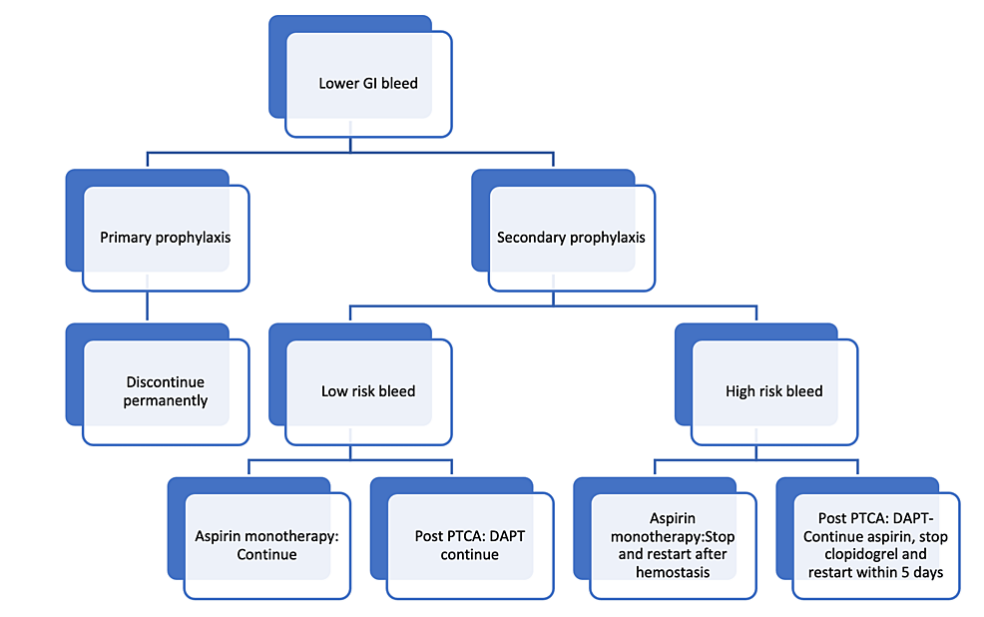
Management of lower gastrointestinal bleed in a patient on antiplatelet therapy PTCA: percutaneous transluminal coronary angioplasty; DAPT: dual antiplatelet therapy

Apart from upper gastrointestinal bleeding, antiplatelet agents can also cause lower gastrointestinal bleeding. In a 10-year prospective study by Ray et al., lower gastrointestinal bleed contributed to 45% of cases of antiplatelet-induced gastrointestinal bleeding. Amongst cases of lower gastrointestinal bleed, the most common source was the small intestine (84%) followed by the colon (16%) [26]. The following flowchart may guide decision-making regarding antiplatelet therapy in lower gastrointestinal bleeding [27] (Figure 1).

Restarting antiplatelet agent

Recovery of platelet aggregation function after stoppage of antiplatelet agent occurs faster with aspirin (four days) compared to clopidogrel (10 days) [28,29]. While reintroducing the antiplatelet on the basis of indication, one has to think of the same two points that were considered while stopping the agents - an indication of antiplatelet therapy and severity of the bleed.

Regarding monotherapy, if the patient was on clopidogrel, then they should switch to aspirin which has a relatively lower risk of bleed. If the patient was on aspirin then to restart the same agent [19], one must consider various factors to reduce the risk of bleeding in the future [30-32] (Table 6).

**Table 6:** Strategies to reduce the risk of rebleeding

Strategies to reduce the risk of rebleed	
Number of agents	To review the indication for dual antiplatelet therapy
Dosage	Start with a lower dose and gradually escalate to the target dose
Frequency	Alternate day dosing followed by daily dosage
Drug interactions	Consider drug interactions that can potentially increase the dose of antiplatelet in the future
Proton pump inhibitor cover	While administering proton pump inhibitors, it is also important to consider interaction with clopidogrel that may reduce the efficacy of Clopidogrel. Clopidogrel is a prodrug that is converted to its active form in the liver by the CYP2C19 enzyme and is inhibited by proton pump inhibitors. The extent of inhibition of enzymes is as following: omeprazole>pantoprazole>lansoprazole>rabeprazole. Interaction can be reduced by changing the timing of administration with PPI given early morning and clopidogrel at bedtime
H.pylori	Testing for H.pylori and eradication
Patient education	Patient awareness regarding signs of bleed like dark-coloured stools and early presentation to the nearest healthcare facility
Dual antiplatelet therapy	In the case of dual antiplatelet therapy, prefer clopidogrel over ticagrelor as a second agent

While prescribing antisecretory agents along with antiplatelet agents, proton pump inhibitors are preferred over H2 receptor antagonists. H2 receptor antagonists were not found to be very effective at preventing clopidogrel-induced gastrointestinal bleeding as compared to proton pump inhibitors [33]. 

If the patient was on dual antiplatelet therapy, then it is of utmost importance to review the indication for a second antiplatelet agent. There are several instances that occur post-coronary stenting and post-ischemic cerebrovascular accidents where the patient is on a second antiplatelet agent beyond recommended duration. Some recommend switching from dual antiplatelet therapy to a single agent after three months of percutaneous intervention for those at high risk of gastrointestinal bleeding, provided the patient is at low risk of an ischemic event [34].

H. pylori eradication plays role in primary as well as secondary prevention of antiplatelet-induced bleeding. A randomised control trial by Hawkey et al. tested the efficacy of H. pylori eradication in preventing aspirin-associated gastrointestinal bleeding. It was noted that H. pylori eradication helped reduce the occurrence of aspirin-induced gastrointestinal bleeding, however, the effect was found to last for two and half years on follow-up and not beyond that. It would be ideal to test for H. pylori prior to initiating an antiplatelet agent for the long term in every case [35].

Scoring systems can be used to assess the risk of gastrointestinal bleeding in those on antiplatelet agents. The (ABC)_2_D score is one such scoring system. Scoring systems are necessary to predict the events of gastrointestinal bleeding. It also helps the physician in giving a prognosis based on the risk score. However, it has not been used on a large scale. It is based on seven factors (i.e., an age of >65 years, anaemia, recent major bleeding, a history of gastrointestinal bleeding, no-proton pump inhibitors use, combined use of oral anticoagulants, and dual antiplatelet therapy [5].

## Conclusions

Antiplatelet agents are used worldwide on a long-term basis and it is imperative to know the potential adverse effect of gastrointestinal bleeding and the management of the same. Broadly speaking, it is the indication of antiplatelet therapy and the severity of the bleed that have to be considered while managing antiplatelet-induced bleeds. Several strategies like proton pump inhibitor cover, eradication of H. pylori, and consideration of drug interactions should be considered to prevent antiplatelet-induced gastrointestinal bleeding in the first place. Aspirin plus PPI would be preferred over clopidogrel or clopidogrel plus PPI .

In a nation like India where a lot of medications are available, the prescription of antiplatelet agents is to be regulated and monitored. Dosing and duration of antiplatelet therapy are to be reviewed at each visit. Patient awareness is also of utmost importance considering the widespread use of antiplatelet therapy and the mortality risk associated with antiplatelet therapy-induced gastrointestinal bleeds.
